# Multi-copy alpha-amylase genes are crucial for *Ditylenchus destructor* to parasitize the plant host

**DOI:** 10.1371/journal.pone.0240805

**Published:** 2020-10-26

**Authors:** Ling Chen, Mengci Xu, Chunxiao Wang, Jinshui Zheng, Guoqiang Huang, Feng Chen, Donghai Peng, Ming Sun

**Affiliations:** State Key Laboratory of Agricultural Microbiology, College of Life Science and Technology, Huazhong Agricultural University, Wuhan, China; Assam Agricultural University Faculty of Agriculture, INDIA

## Abstract

*Ditylenchus destructor* is a migratory plant-parasitic nematode that causes huge damage to global root and tuber production annually. The main plant hosts of *D*. *destructor* contain plenty of starch, which makes the parasitic environment of *D*. *destructor* to be different from those of most other plant-parasitic nematodes. It is speculated that *D*. *destructor* may harbor some unique pathogenesis-related genes to parasitize the starch-rich hosts. Herein, we focused on the multi-copy alpha-amylase genes in *D*. *destructor*, which encode a key starch-catalyzing enzyme. Our previously published *D*. *destructor* genome showed that it has three alpha-amylase encoding genes, *Dd_02440*, *Dd_11154*, and *Dd_13225*. Comparative analysis of alpha-amylases from different species demonstrated that the other plant-parasitic nematodes, even *Ditylenchus dipsaci* in the same genus, harbor only one or no alpha-amylase gene, and the three genes from *D*. *destructor* were closely clustered in the phylogenetic tree, indicating that there was a unique expansion of the alpha-amylase gene in *D*. *destructor*. The enzymatic activity of the three alpha-amylase proteins was verified by an enzyme assay. Quantitative real-time PCR assay showed that the expression of the three alpha-amylase genes in the post-hatching stage of *D*. *destructor* was found to be significantly higher than that in eggs. In the *in situ* hybridization assay, the expression of the genes was localized to the intestine, implying the association of these genes with nematode digestion. An infection assay in sweet potato demonstrated that RNA interference of any one alpha-amylase gene had no influence on the infectivity of *D*. *destructor*. Using the multi-target dsRNA cocktail method, it was found that silencing of two of the three genes inhibited nematode infection, and the infectivity of worms treated with three dsRNA simultaneously changed the most, which decreased by 76.6%. Thus, the multi-copy alpha-amylase genes in *D*. *destructor* are compensatory and crucial for nematodes to parasitize the plant host.

## Introduction

Over 4100 species of plant-parasitic nematodes are distributed globally and cause huge agricultural losses ($80~173 billion) annually [1–3]. Among them, *Ditylenchus destructor* Throne, commonly known as potato rot nematode or stem nematode, is one of the primary pathogens in the global production of sweet potato (*Ipomoea batatas*) and potato (*Solanum tuberosum*), the two main root and tuber crops essential for food security [4, 5]. In China, the country producing 75% production of world sweet potato, *D*. *destructor* causes significant yield losses of 20% to 50%; in endemic regions, the losses may reach 100% [6]. Except for sweet potatoes and potatoes, the hosts of *D*. *destructor* are related to a wide range of plants and fungi [7]. These nematode individuals penetrate plant organs and cause separation of the host cells, eventually resulting in plant rot [8]. Currently, the main strategies for controlling *D*. *destructor* are chemical nematicides, crop rotation, and resistant crop varieties. However, these strategies have certain limitations: most chemical nematicides are unfriendly to the environment, crop rotation fetches less profit to farmers, and the number of available resistant crop varieties is limited [9]. Hence, it is necessary to explore an effective and safe strategy against *D*. *destructor*. However, the inadequate understanding of the nematode parasitic mechanism limits the development of nematode control strategies.

In the process of studying the parasitic mechanism of plant-parasitic nematodes, research has found that these nematodes harbor a series of pathogenesis-related genes to facilitate parasitizing the hosts [10]. These genes include not only the genes encoding the effector proteins secreted by the esophageal glands, but also those related to nematode infection [11]. The functions of the pathogenesis-related genes are related to the penetration, migration, and maintenance in the host of plant-parasitic nematodes [12]. Studies on pathogenesis-related genes are not only effective for revealing the parasitic mechanism of plant-parasitic nematodes, but also provide target resources for using RNA interference (RNAi) to control nematodes. RNAi is an important reverse genetic tool for studying gene function in plant-parasitic nematodes and can be a potential strategy for nematode control [13]. The exogenous double-stranded RNA (dsRNA) and small interfering RNA (siRNA) ingested by nematodes can cause the silencing of homology-dependent endogenous genes through the conserved RNAi pathway in nematodes [14]. Therefore, expressing dsRNA or siRNA targeting nematode pathogenesis-related genes in plant cells can improve plant resistance against plant-parasitic nematodes [15]. Currently, most studies on pathogenesis-related genes are focused on the sedentary root-knot nematode (*Meloidogyne* spp.) and cyst nematode (*Heterodera* and *Globodera* spp.) for their destructive impact on global agriculture. In contrast, studies on the pathogenesis-related genes in *D*. *destructor* are relatively few [9, 16]. *D*. *destructor* is a migratory plant-parasitic nematode, and the lifestyle and parasitic environment are quite different from those of sedentary nematodes. Therefore, even though the parasitic mechanisms reported in sedentary nematodes could serve some useful guidance in studies of *D*. *destructor*, it is still not enough to uncover the mystery of *D*. *destructor* parasitism.

The main plant hosts of *D*. *destructor*, sweet potatoes and potatoes, contain plenty of starch in their storage roots or tubers. This characteristic makes the parasitic environment of *D*. *destructor* different from those of most other plant-parasitic nematodes. To successfully parasitize the starch-rich hosts, it is speculated that *D*. *destructor* may harbor some unique pathogenesis-related genes. During the genome sequencing project of *D*. *destructor*, we found that the alpha-amylase gene (EC 3.2.1.1), encoding one of the key starch-converting enzymes, is slightly expanded [17]. Alpha-amylase belongs to the family of 13 glycosyl hydrolases, and can hydrolyze starch by cleaving to the alpha-1,4 glycosidic bonds of the inner part (endo-) of the amylose or amylopectin chain [18]. It is hypothesized that the triple-copy alpha-amylase genes are the pathogenesis-related genes of *D*. *destructor* which facilitate the parasitizing of hosts by the nematodes. Nevertheless, whether the alpha-amylase genes truly play a crucial role during plant-parasitic nematode infection is not identified. In view of this, the present study describes the characterization of the three alpha-amylase genes (Gene ID in WormBase ParaSite Database: *Dd_02440*, *Dd_11154*, and *Dd_13225*) in *D*. *destructor* by using sequence analysis and enzyme assays. The temporal and spatial expression of the three genes was explored by quantitative real-time PCR (qRT-PCR) and *in situ* hybridization. With infection assays based on *in vitro* RNAi, the crucial role of each of the alpha-amylase genes in *D*. *destructor* infection was detected.

## Materials and methods

### Nematode culture and collection

*D*. *destructor* Dd01 was isolated from infected sweet potatoes in Wuhan, China, and was maintained in the storage roots of sweet potato [17]. The sweet potato storage roots were washed with soapy water and clean water, sterilized with 95% ethanol, and finally treated with UV for 30 min after drying. Approximately 1000 sterilized nematodes were inoculated into a hole in the storage root, which was dug using a sterile scalpel. The hole was then filled with sweet potato tissue, which was dug out, and sealed with paraffine. The inoculated storage roots were incubated at 25°C in the dark. After four or five weeks, the inoculated storage roots were cut into pieces with a sharp kitchen knife, and the nematodes in mixed stages (egg, larva, female and male adults) were extracted from the inoculated sweet potatoes using the modified Baermann method [19]. The extracted nematodes were soaked in a 0.5% NaOCl solution for 2 min for surface sterilization and washed with sterile water three times. For the qRT-PCR assay, eggs were screened out from the mixed stage nematodes with a 900 mesh sieve, and J2 were molted from the screened eggs. The nematodes at other developmental stages (J3, J4, male and female) were carefully picked out from the extracted nematodes respectively with a stereomicroscope (SZX16, Olympus).

### Comparison of alpha-amylases from animal species

The alpha-amylases in *D*. *destructor* were compared with those of other nematodes (including free-living, plant-parasitic, and animal-parasitic nematodes), insects, and some higher animals. The amino acid sequences of alpha-amylase proteins in different species were obtained from the available genomic data in the NCBI database (https://www.ncbi.nlm.nih.gov) and the Wormbase ParaSite database (http://parasite.wormbase.org/index.html) according to the bioinformatics methods described previously [17]. These sequences were aligned using Muscle [20] with default settings. The alignment was trimmed using trimAl with an automated method [21]. A maximum-likelihood phylogenetic tree of alpha-amylase was constructed based on the alignment, using Mega-X bioinformatics tool [22]. Bootstrap support values were calculated from 500 replicates.

### RNA extraction and cDNA synthesis

The total RNA of *D*. *destructor* was extracted by using *TranZol*^TM^ Up Plus RNA Kit (TransGen Biotech) according to the manufacturer’s protocol, and the quality of RNA was detected by gel electrophoresis and NanoDrop 2000 (Thermo Fisher). First-strand cDNA was generated using PrimeScript^TM^ RT reagent Kit with gDNA Eraser (TaKaRa) according to the manufacturer’s protocol.

### Heterologous expression of alpha-amylase proteins from *D*. *destructor*

The three alpha-amylase genes (*Dd_02440*, *Dd_11154*, and *Dd_13225*) were amplified from cDNA using ExTaq polymerase (TransGen Biotech) and the designed primer (S1 Table). The PCR products were inserted into the multiple cloning site of the expression vector pET28a respectively to express a His-tagged protein in *Escherichia coli* BL21 (DE3). The expression of the alpha-amylase proteins was conducted according to a method described by Liu [23]. The target proteins were purified from recombinant *E*. *coli* with His-Bind columns (Qiagen, Germany), according to the manufacturer’s protocol. The purified proteins were detected by sodium dodecyl sulfate-polyacrylamide gel electrophoresis (SDS-PAGE) and western blotting according to the method described by Li [24].

### Enzyme activity assay of alpha-amylase

The activities of the purified alpha-amylases, Dd_02440, Dd_11154, and Dd_13225, were assayed as described by Yoo [25], with some modifications. To determine the effect of temperature on the enzyme activity of alpha-amylase, reactions were carried out at 20°C, 40°C, 60°C, and 80°C. To detect the effect of pH on alpha-amylase activity, sodium citrate buffer (pH 5.0), phosphate buffer solution (pH 7.4), and sodium carbonate-sodium bicarbonate buffer (pH 9.6) were used, and the reactions were carried out at 60°C. The proteins and reagents were preheated in a water bath at the corresponding temperature before the experiment. 25 μg tested protein was added to 200 μL of 0.5% starch solution, and the total volume of the solution was set to 500 μL. After 10 min of treatment at the corresponding temperature, 50 μL of 0.1 M hydrochloric acid solution was added to the solution to terminate the reaction. Then, 2 μL of 0.1 M iodine solution was added, and the total volume of the solution was set to 4 mL. The negative control was the reaction solution without protein and starch, and the positive control was the reaction solution without protein. Using a spectrophotometer (Spectrumlab 23A, Lengguang Technology, China), the intensity of the reaction mixture was measured at OD620 using the negative control as a substrate. One unit of enzyme activity was expressed as the amount of alpha-amylase to degrade 1 μM of starch per minute at 60°C.

### Analysis of alpha-amylase genes expression in each developmental stage of *D*. *destructor*

The total RNA of eggs and J2 were extracted from approximately 10,000 eggs and 3,000 J2 individuals, respectively. The total RNA from the other stages (includingJ3, J4, male, and female) was extracted from approximately 100 nematodes. The total RNA of different stages was reverse transcribed to cDNA respectively. All the primers used for qRT-PCR (S1 Table) had been designed and optimized for working concentration and annealing temperature. The qualitative analysis of gene expression in nematodes was performed with the SYBR^®^ Green Realtime PCR Master Mix (TOYOBO Corporation, Japan) according to the manufacturer’s protocol. Gene expression was normalized with a nematode-conserved alpha-tubulin gene *Dd-tba-1* (Gene ID in WormBase ParaSite Database: *Dd_06531*) as an internal reference [26]. All PCRs were performed in triplicate and the mean Ct values were determined. Relative gene expression was determined using the 2^-ΔΔCt^ method as described in the ABI PRSIM Vii7 Sequence Detection System (Applied Biosystems).

### *In situ* hybridization of the transcripts of alpha-amylase genes in *D*. *destructor*

To localize the expression of the transcripts of the three alpha-amylase genes (*Dd_02440*, *Dd_11154*, and *Dd_13225*) in *D*. *destructor*, *in situ* mRNA hybridization was performed. The cDNA fragments of the three genes were, respectively, amplified from their recombinant expression vector using the designed primers (S1 Table). The PCR products were used to synthesize the digoxigenin (DIG)-labeled sense and antisense cDNA probes by asymmetric PCR with either forward or reverse primers using the DIG-labelling Kit (DDLK-010, MyLab^TM^). Nematode fixation, permeabilization and detection were performed according to the methods described by Peng [16] and the manufacturer’s protocol of the DIG hybridization detection Kit I (DIGD-110, MyLab^TM^). Nematode specimens were mounted on glass slides and examined using a motorized microscope (BX63, Olympus).

### RNAi of alpha-amylase genes in *D*. *destructor*

The DNA templates for synthesizing double-stranded RNA (dsRNA) corresponding to the three alpha-amylase genes were amplified from the cDNA with *EasyTaq* DNA polymerase (TransGen Biotech) and designed primers (S1 Table). The DNA templates of the non-endogenous gene *gfp* encoding green fluorescent protein were amplified using vector pHT315-GFP as a PCR template. The DNA templates were transcribed into single-strand RNA (ssRNA) firstly by a using T7 *in vitro* Transcription Kit (Cat No. BK0034, Biomics) according to the manufacturer’s protocol, and then the ssRNA was annealed into dsRNA by incubating it in boiling water for 5 min and subsequently at room temperature for 1 h. The DNA templates were removed by DNase I (TaKaRa) treatment, then the dsRNA was extracted using phenol: choloroform: isoamyl alcohol (25: 24: 1) and precipitated with ethanol. Finally, the precipitated dsRNA was dissolved in RNase-free water. The quality of dsRNA was detected by gel electrophoresis and NanoDrop 2000 (Thermo Fisher). Using 1 mg/mL Fluorescein isothiocyanate isomer-I (FITC) (Sigma) as a marker, we found that the optimal nerve stimulant to stimulate nematodes ingesting soaking solution was 50 mM octopamine hydrochloride and 3 mM spermidine (S1 Fig). Approximately 500 worms at mixed stages were soaked in 1000 mg/mL target dsRNA in 50 μL soaking solution containing the nerve stimulant and 0.01% TritonX-100 (Sigma). A multi-target dsRNA cocktail method that mixed two or three dsRNA species in the soaking solution was used to knock-down two or three alpha-amylase genes simultaneously. The final concentration of each dsRNA species was 1000 mg/mL. After incubation in the dark on a slowly moving rotator at 25°C for 24 h, the worms were washed four times in sterile water. Nematodes soaked in *gfp* dsRNA solution were used as the negative controls. qRT-PCR was performed as described before to detect the mRNA abundance of the three alpha-amylase genes after RNAi.

### Infection assay of RNAi-treated nematodes in the storage roots of sweet potato

To assess the post-RNAi effect of alpha-amylase genes on the infectivity of *D*. *destructor*, the relative infection area and reproduction of RNAi-treated worms in the storage roots of sweet potato were analyzed. Approximately 500 nematodes soaked with dsRNA for 24 h were inoculated into storage roots using the method of artificial inoculation described by Fan [9], with some modifications. The inoculated storage roots were maintained in an incubator in the dark at 25°C. Worms soaked in *gfp* dsRNA solution were also inoculated as the control. All infection assays were performed in five replicates. After 14 days, the relative infection area was calculated as the ratio of the infection area to the total area of the transverse sections of storage roots using ImageJ software [27]. Afterwards, the nematodes were collected from the inoculated storage roots by the method described before, and the nematode number was counted.

### Data analysis

All experiments were performed at least three times. The differences between control and experimental samples were analyzed by one-way ANOVA with Tukey’s HSD test (P < 0.05) using SPSS Statistics v22.0 (IBM). Data were presented using GraphPad Prism 6.

## Results

### Characterization of alpha-amylases in *D*. *destructor*

**Fig 1:**
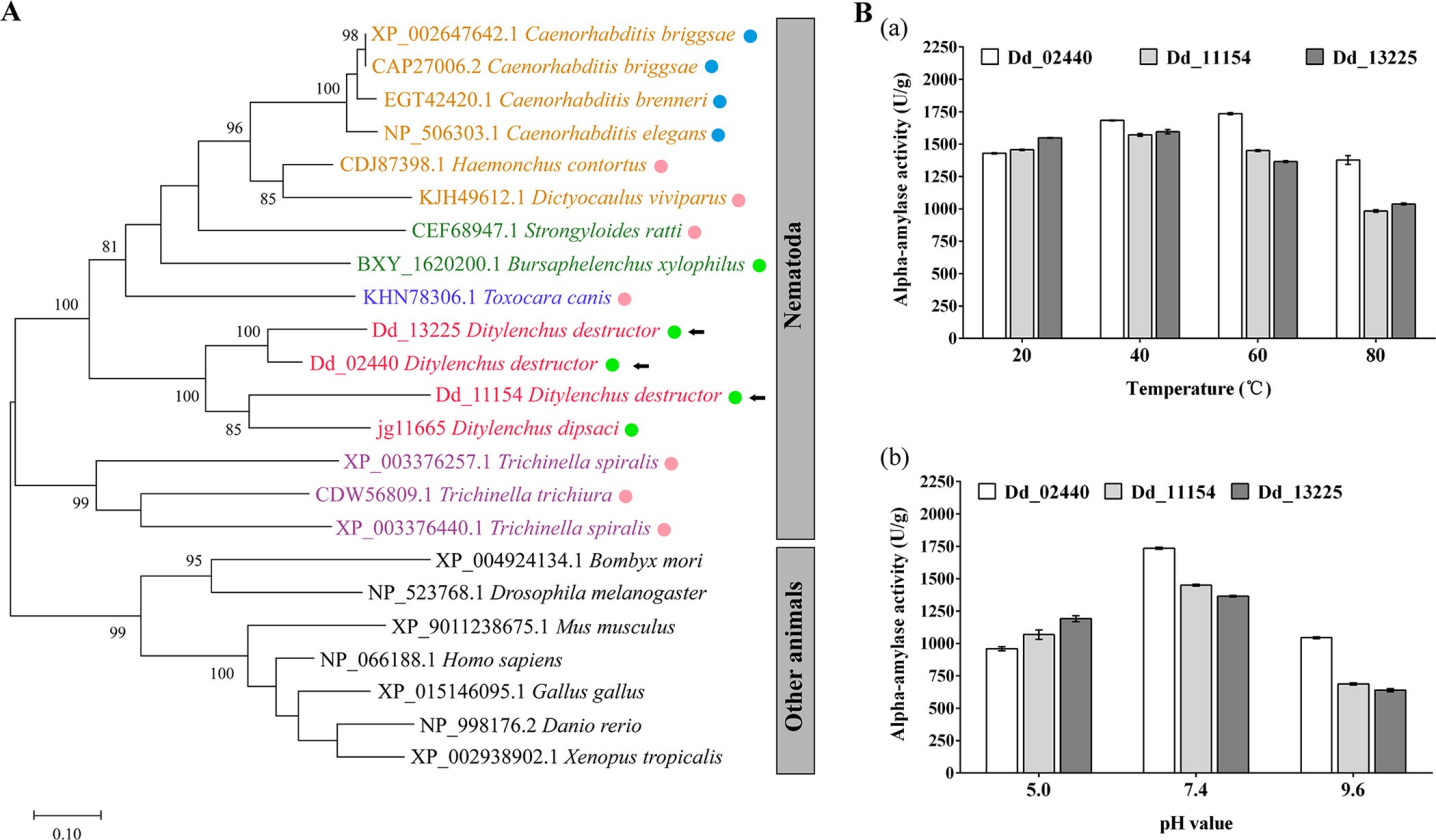
Characterization of three alpha-amylase proteins in *D*. *destructor*. (A) Phylogenetic analysis of alpha-amylase proteins from 12 nematode species with different lifestyles encompassing clades 2, 8, 9, 10, and 12 in phylum Nematoda and several other animals based on the amino acid sequence. Taxon names are colored for different clades in phylum Nematoda (purple for Clade 2, brown for Clade 8, blue for Clade 9, green for Clade 10, and red for Clade 12). The different colors of circles on the right of taxon names represent different feeding behaviors (blue for free-living, pink for animal-parasitism, and green for plant-parasitism). The alpha-amylase proteins in *D*. *destructor* are arrowed. Only bootstrap values above 80 are shown in numbers. (B) Effect of temperatures (a) and pH (b) on the activity of Dd_02440, Dd_11154, and Dd_13225. Each column represents the mean ±standard errors of three repetitions.

To detect the characteristics of alpha-amylase genes in *D*. *destructor*, we first performed a phylogenetic analysis based on the amino acid sequences of alpha-amylase proteins in *D*. *destructor* and several species, including nematodes, insects, and some higher animals. Based on small subunit ribosomal DNA (SSU rDNA) sequences, the phylum Nematoda is divided into twelve clades, among which plant-parasitic nematodes were found in Clade 1 (Triplonchida), 2 (Dorylaimida), 10 (Aphelenchoididae), and 12 (Tylenchida) [28]. Eleven nematode species, representing diverse feeding behaviors and diverse clades in the phylum Nematoda, were chosen. The phylogenetic tree demonstrated that alpha-amylases from nematodes were clustered together, which reveals the inter-clade conservation of alpha-amylase proteins in the phylum Nematoda (Fig 1A). Interestingly, the cluster of alpha-amylase proteins in the genus *Ditylenchus* (belonging to Clade 12) was positioned closer to the root than the nematodes belonging to clades 8, 9, and 10, suggesting that *Ditylenchus* spp. might obtain alpha-amylase genes from a relatively ancient origin. In the analysis process, we noticed that most nematodes harbor only one alpha-amylase gene, even *Ditylenchus dipsaci*, another important plant-parasitic nematode in the genus *Ditylenchus*, has only one alpha-amylase gene (Gene ID in WormBase ParaSite Database: *jg11665*). Some plant-parasitic nematodes, such as *Meloidogyne incognita* and *Globodera pallida* harbor no alpha-amylase genes. The results suggest that the expansion of the alpha-amylase gene is a species-specific characteristic of *D*. *destructor*. Among the three alpha-amylase proteins in *D*. *destructor*, Dd_13225 shares 77.4% sequence identity with Dd_02440 and has 56.3% identity with Dd_11154, Dd_02440 shares 59.2% sequence identity with Dd_11154. The alignment of the amino acid sequence of the three alpha-amylase proteins is shown in S2 Fig. Therefore, we defined the three alpha-amylases as expanded multi-copy alpha-amylases in *D*. *destructor*.

Subsequently, the three predicted alpha-amylase proteins, Dd_02440, Dd_11154, and Dd_13225, were purified from the heterologous expression system of *E*. *coli* and detected by SDS-PAGE and western blotting (S3 Fig). An enzyme activity assay was performed to test amylase activity. All proteins showed relatively good hydrolytic activity toward starch over a broad temperature range from 20°C to 80°C (Fig 1B(a)). The maximum activity value of Dd_02240 were 1735.9 U/g at 60°C, and that of Dd_11154 and Dd_13225 was 1572.3 U/g and 1596.4 U/g, respectively, at 40°C. The optimal pH for the three proteins was shown at pH 7.4 (Fig 1B(b)).

### Stage-specific expression and tissue localization of alpha-amylases in *D*. *destructor*

**Fig 2:**
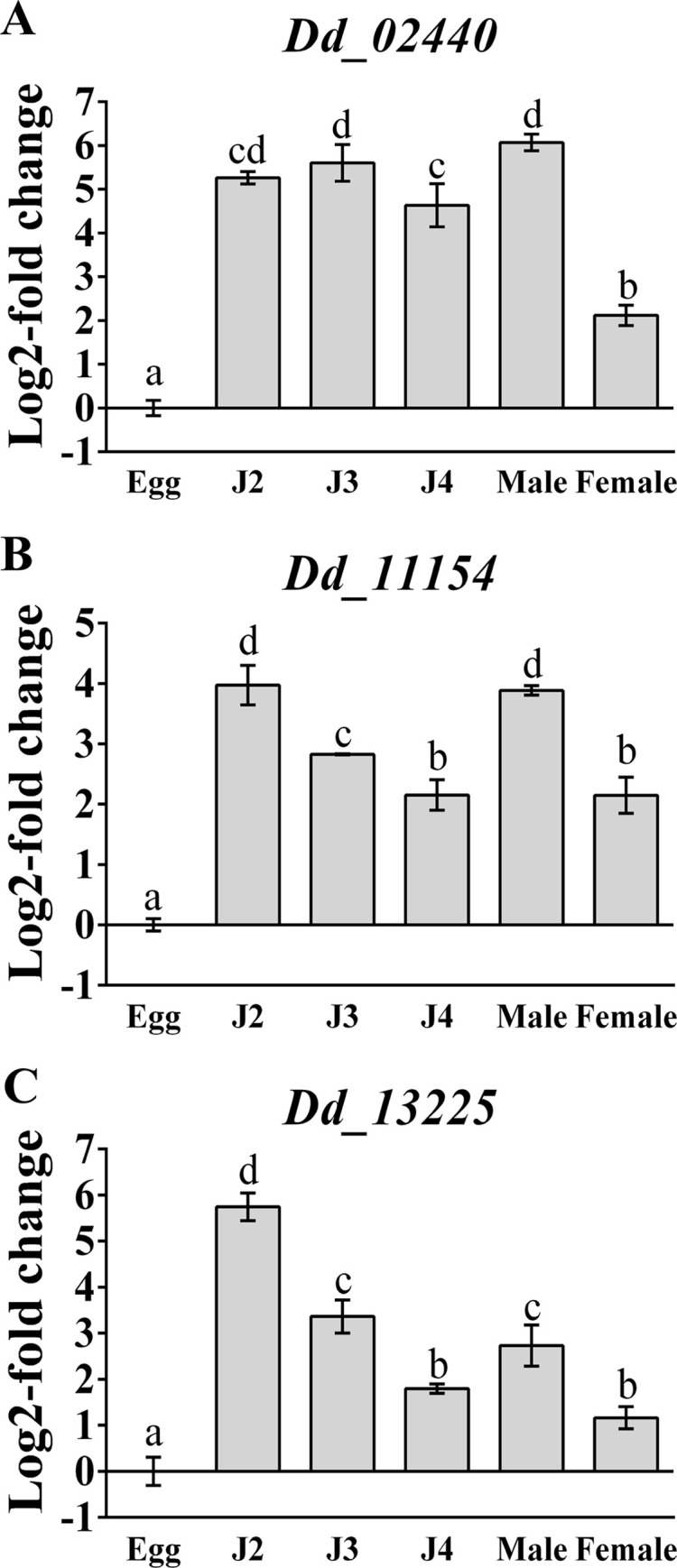
Relative transcript abundance of three alpha-amylase genes in each developmental stage of *D*. *destructor*. By using the transcript level in eggs as a reference, *Dd_02440* (a), *Dd_11154* (b), and *Dd_13225* (c) were significantly up-regulated in other stages (J2, J3, J4, adult males and females). The quantitative RT-PCR values are means ± standard errors for three biological replicates. Letters indicate significant differences according to one-way ANOVA with Tukey’s HSD tests (P < 0.05).

The specific expression of the three alpha-amylase genes in each developmental stage of *D*. *destructor* was detected by qRT-PCR. By using the expression level in eggs as a reference, the expression levels of *Dd_02440* in the other five stages (J2, J3, J4, male, and female) were respectively up-regulated by 5.26 ± 0.14, 5.60 ± 0.42, 4.63 ± 0.49, 6.07 ± 0.19, and 2.12 ± 0.23-fold significantly on a log_2_ scale (Fig 2(A)). *Dd_11154* was also significantly up-regulated by 3.97 ± 0.33, 2.83 ± 0.01, 2.15 ± 0.25, 3.89 ± 0.08, and 2.15 ± 0.30-fold in the other five stages on a log2 scale, respectively (Fig 2(B)). Similarly, the expression of *Dd_13225* was up-regulated by 5.75 ± 0.30, 3.36 ± 0.36, 1.80 ± 0.10, 2.73 ± 0.45, and 1.16 ± 0.24-folds, respectively (Fig 2(C)). These data demonstrated that all three genes were highly expressed during the whole post-hatching stage of *D*. *destructor*, implying the important role of alpha-amylase in hatched worms.

**Fig 3:**
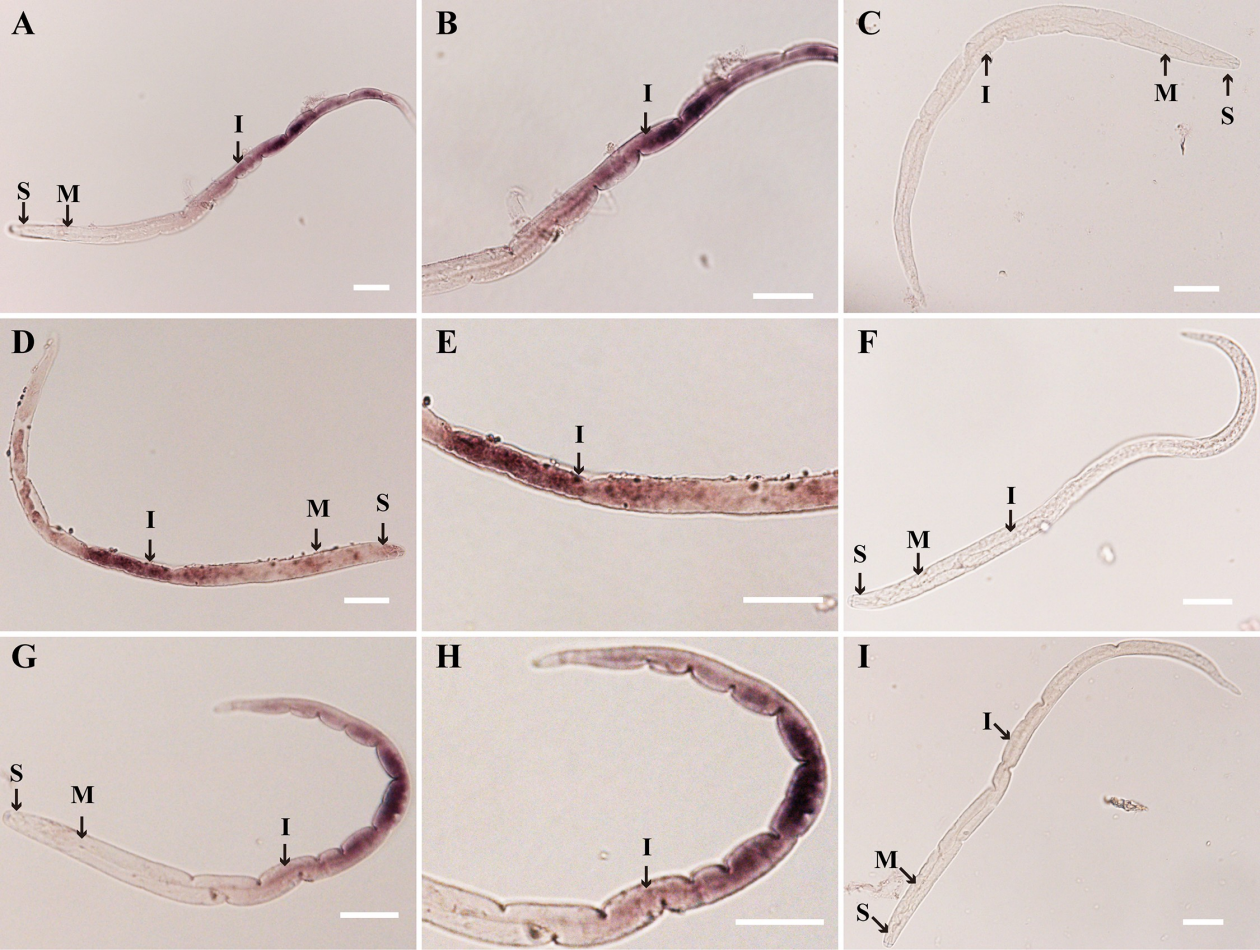
Location of the transcripts of alpha-amylase genes in the intestine of *D*. *destructor* by *in situ* hybridization. The site of gene expression is signified by black color due to the enzymatic cleavage of a chromogenic substrate by alkaline phosphatase-conjugated to the anti-DIG antibody. The DIG-staining in nematodes showed specific binding of the antisense probes of *Dd_02440* (A), *Dd_11154* (D), and *Dd_13225* (G) to the intestine. Photograph (B), (E), and (H) are the magnified views of (A), (D), and (G) respectively. No staining was observed with *Dd_02440* (C), *Dd_11154* (F), and *Dd_13225* (I) sense probes as the negative control. I represents intestine; M represents median bulb; S represents stylet. Scale bar = 20 μm.

In the *in situ* hybridization assay, the tissue locations of the three genes in *D*. *destructor* were detected. Nematodes were processed for *in situ* hybridization with antisense and sense DIG-labeled DNA probes specific to *Dd_02440*, *Dd_11154*, and *Dd_13225*, respectively. DIG staining of the hybridization with three antisense probes revealed that the three alpha-amylase genes appeared to be expressed in the intestine of *D*. *destructor* (Fig 3A, 3B, 3D, 3E, 3G and 3H). Hybridization with the sense probes showed no results in staining and were set as the negative control (Fig 3C, 3F and 3I). The expression location of the three genes suggested that the function of alpha-amylase in *D*. *destructor* may be associated with nematode digestion.

### RNAi of at least two alpha-amylase genes impedes the infectivity of *D*. *destructor* on sweet potato

**Fig 4:**
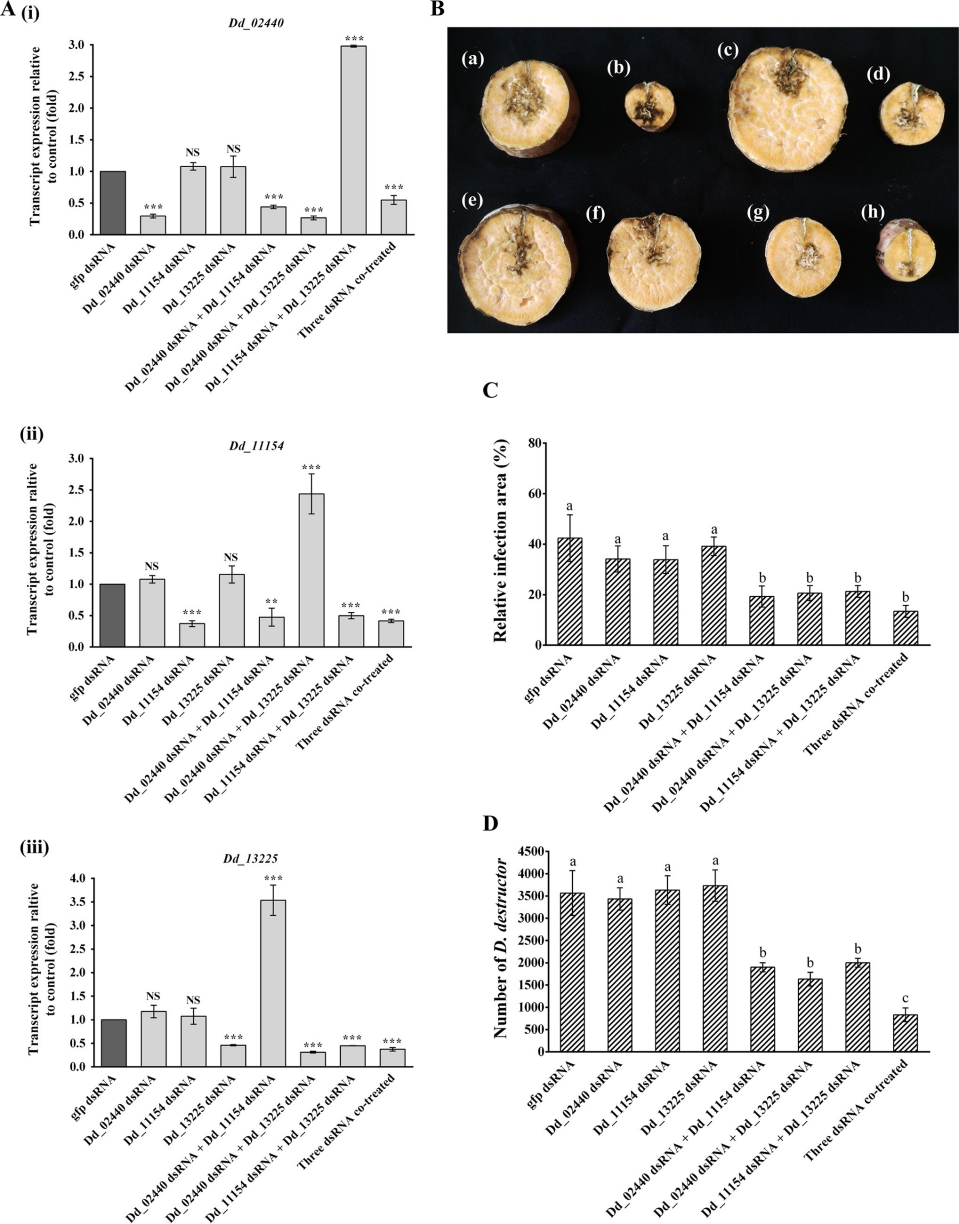
Effect of *in vitro* silencing of the three alpha-amylase genes on the infectivity of *D*. *destructor*. (A) RNAi effect of different dsRNA soaking solutions on the transcript abundance of *Dd_02440* (i), *Dd_11154* (ii), and *Dd_13225* (iii) in *D*. *destructor* mixed stages at 24 h. Control worms were soaked in the soaking solution with non-target *gfp* dsRNA. Fold change in the expression of the three alpha-amylase genes relative to the reference transcript of *Dd-tba-1*, a nematode-conserved gene encoding alpha-tubulin, was quantified using the 2^-ΔΔCt^ method. Quantitative RT-PCR values are means ± standard errors for three biological replicates. Asterisks on top of the bars indicate significant differences according to one-way ANOVA with Tukey’s HSD tests (**P < 0.01 and ***P < 0.001), and NS indicates no significant difference. (B) The extent of the rot infected areas in the transverse sections of storage roots inoculated with different dsRNA-treated nematodes at 14 dpi (days post-inoculation): (a) nematodes treated with *gfp* dsRNA, (b) nematodes treated with *Dd_02440* dsRNA, (c) nematodes treated with *Dd_11154* dsRNA, (d) nematodes treated with *Dd_13225* dsRNA, (e) nematodes treated with *Dd_02440* dsRNA and *Dd_11154* dsRNA, (f) nematodes treated with *Dd_02440* dsRNA and *Dd_13225* dsRNA, (g) nematodes treated with *Dd_11154* dsRNA and *Dd_13225* dsRNA, and (h) nematodes treated with *Dd_02440* dsRNA, *Dd_11154* dsRNA, and *Dd_13225* dsRNA. (C) The relative infection areas in the storage roots infected with different dsRNA-treated nematodes at 14 dpi. (D) The colonization numbers of *D*. *destructor* in the storage roots infected with different dsRNA-treated nematodes at 14 dpi. Nematodes treated with g*fp* dsRNA were set as the control. In (C) and (D), each column represents the mean ± standard errors of five repetitions. Letters indicate significant differences according to one-way ANOVA with Tukey’s HSD tests (P < 0.05).

In order to determine the role of alpha-amylase in the infectivity of *D*. *destructor*, RNAi soaking experiments were performed. Three dsRNA species were designed to target the corresponding alpha-amylase genes specifically. Given that the stage-specific expression and tissue location among *Dd_02440*, *Dd_11154*, and *Dd_13225* are similar, we propose that the three alpha-amylase genes may be functionally redundant for *D*. *destructor*. Therefore, a multi-target dsRNA cocktail method was used to detect changes in nematode infectivity when two or three alpha-amylase genes were silenced simultaneously [29]. The post-RNAi changes in the transcriptional levels of the three alpha-amylase genes were measured by qRT-PCR compared with worms soaked in non-target *gfp* dsRNA. When the worms were treated with only one dsRNA species, the expression of the corresponding gene was down-regulated significantly, while the other two genes showed no significant change; RNAi with any of the two dsRNA species decreased the expression of the two corresponding genes, however, the expression of the other one gene was upregulated; the expression of the three genes in worms soaked with three dsRNA species were all down-regulated significantly (Fig 4A). The upregulation of one alpha-amylase gene when the other two genes were knocked down suggested that there may be a compensatory mechanism among the three genes.

An infection assay was conducted to detect the effect of RNAi of alpha-amylase genes on the infectivity of *D*. *destructor* in the sweet potatoes. Worms treated with different dsRNA solutions were inoculated in the storage roots of sweet potato. At 14 dpi, the infection areas were observed surrounding the inoculation site in the transverse sections of the storage roots (Fig 4B). The relative infection areas in the inoculated storage roots were calculated. The infection area of the nematodes treated with only one alpha-amylase gene RNAi showed no significant changes compared with those of the nematodes treated with non-target *gfp* dsRNA. However, the silencing of two or three alpha-amylase genes decreased the nematode infection area (Fig 4C). Subsequently, nematodes were collected from the inoculated sweet potatoes and counted. Compared with the nematodes treated with *gfp* dsRNA, the single RNAi of *Dd_02440*, *Dd_11154*, and *Dd_13225* also showed no significant influence on the reproduction of *D*. *destructor* in sweet potato. When two of the three alpha-amylase genes were silenced, worms displayed significantly reduced reproduction by 46.7% (co-RNAi of *Dd_02440* and *Dd_11154*), 54.2% (co-RNAi of *Dd_02440* and *Dd_13225*), and 43.9% (co-RNAi of *Dd_11154* and *Dd_13225*). The colonization number of nematodes treated with the three dsRNA species simultaneously decreased by 76.6% (Fig 4B). These results show that the silencing of at least two alpha-amylase genes inhibits the infectivity of *D*. *destructor* on sweet potato.

## Discussion

Infected sweet potatoes or potatoes can release a large amount of starch due to the destructive infection caused by *D*. *destructor*; this starch might be selected first as the carbohydrate resource for growth by nematodes. Since starch can be metabolized and utilized by organisms only with the action of starch-converting enzymes [30], we proposed a hypothesis that the multi-copy alpha-amylase genes are crucial for *D*. *destructor*’s parasitization of starch-rich hosts. Hence, the present study focuses on the molecular and functional characterization of the three alpha-amylase genes (*Dd_020440*, *Dd_11154*, and *Dd_13225*) in *D*. *destructor*. Based on the biochemical function and the expression location of the three genes, it was shown that the multi-copy alpha-amylases in *D*. *destructor* are not effectors, which are secreted from nematode esophageal glands to interact with plant proteins for disease progression [12]. However, the results of the infection assay demonstrated that when at least two alpha-amylase genes were silenced, the infection area and reproduction of *D*. *destructor* were decreased significantly, indicating that the alpha-amylase genes are crucial for *D*. *destructor* to parasitize the plant host. It was speculated that these three genes might be involved in the nutritional absorption of *D*. *destructor* in the plant host.

When only one alpha-amylase gene was expressed (the other two genes were silenced), the infectivity of *D*. *destructor* in sweet potato decreased significantly (Fig 4), inferring that only one alpha-amylase gene is not enough for *D*. *destructor* to parasitize the host successfully. This may also explain why the expansion of the alpha-amylase gene does not occur in the genome of other plant-parasitic nematodes, such as *D*. *dipasaci* and *Bursaphelenchus xylophilus*, even in the genomes of *M*. *incognita* and *G*. *pallida*, there are no alpha-amylase genes. The main plant organs infected by these plant-parasitic nematodes are not starch-rich. It seems that two alpha-amylase genes are enough for *D*. *destructor* to parasitize the host, however, the fact is that *D*. *destructor* keeps three alpha-amylase genes. In addition, results of the *in situ* hybridization assay also showed that the expression locations of the three genes in *D*. *destructor* were not completely the same, the *Dd_11154* probe-treated nematodes showed a wider stained area than the other two probe-treated nematodes (Fig 3). This result implies that the functions of the three alpha-amylase genes might be different in the intestine of *D*. *destructor*. Probably, due to the limitation of the sensitivity of the infection assay, the biological significance of the three alpha-amylase genes in *D*. *destructor* has not been fully revealed.

Besides the alpha-amylase gene in *D*. *destructor*, some important pathogenesis-related proteins are also encoded by multi-copy genes in some plant-parasitic nematodes. For example, *Globodera rostochiensis* harbors four *eng* genes encoding β-1,4-endoglucanase to facilitate nematode degradation of the hosts’ cell wall [31]; *Heterodera schachtii* secretes two venom allergen-like proteins (Hs-VAP1 and Hs-VAP2) to modulate the activation of basal innate immunity of the host [32]; the chorismate mutases of *Meloidogyne* spp., which alter the host’s cell development are encoded by two genes [33]. These cases imply that gene duplication might be an evolutionary strategy for plant-parasitic nematodes to adapt to the parasitic life in the hosts by increasing the dosage effects or neofunctionalization of key genes.

In this study, the silencing of three genes simultaneously in *D*. *destructor* was achieved using the multi-target dsRNA cocktail method. It has recently been reported that due to the compensatory mechanism of multiple related genes, which often complicates the verification of gene functions [34], multi-copy genes are usually removed from the candidate parasitic genes during the process of bioinformatics prediction [35]. In *D*. *destructor*, with the three target-specific dsRNA species, we also found that there were certain complementary effects among the three alpha-amylase genes. The application of multi-target RNAi is not only conducive to the study of multi-copy genes, but also to the functional location of genes in signal pathways [36]. In the future, more details of the effect of multi-target RNAi in plant-parasitic nematodes need to be studied, especially regarding the stability and the maximum number of targets.

As the main enzyme involved in carbohydrate metabolism, alpha-amylase is widely distributed in animals, plants and microorganisms [37]. In the phylum Nematoda, genome analysis showed that alpha-amylase genes are present in many animal-parasitic and free-living nematodes, and only a few plant-parasitic nematodes [17]. Unlike in *D*. *destructor*, alpha-amylases in animal-parasitic nematodes were reported to be involved in glycogen catabolism [38]. The host environments of other plant-parasitic nematodes that harbor alpha-amylase genes, such as *D*. *dipsaci* and pine wood nematode (*B*. *xylophilus*), are not all starch-rich. Whether the alpha-amylase genes in these plant-parasitic nematodes are also related to nematode pathogenesis needs to be further explored.

As RNAi is a promising strategy for the control of plant-parasitic nematodes, the targets should be designed rationally to avoid off-target effects [39]. Based on the bioinformatic analyses, the alpha-amylase genes in *D*. *destructor* are phylogenetically relatively distant to the alpha-amylase genes in insects and higher animals (Fig 1A). Hence, alpha-amylase genes have the potential to be bio-safe candidate targets against *D*. *destructor*. However, due to the compensatory mechanism among the three alpha-amylase genes, the dsRNA or siRNA expressed in transgenic crops should be designed more carefully. To solve this problem, it may be possible to express three siRNAs targeting the three alpha-amylase genes, or to express one siRNA targeting the homologous fragments of the three genes in plants. In the future, our effort could be focused on the development of RNAi-based transgenic plants to protect the root and tuber crops against *D*. *destructor*.

## Supporting information

S1 Fig FITC fluorescence of *D*. *destructor* incubated for 24 h in soaking solutions with different nerve stimulants. Fluorescence was present in the intestine of nematodes soaked with 50 mM octopamine hydrochloride and 3 mM spermidine. Fluorescence was only present in the stylet of nematodes soaking with 1% resorcinol occasionally, and no fluorescence was observed in nematodes soaked in control solution without any stimulants. The top and middle panels show the photographs of a single nematode on a bright field and under UV, respectively. The bottom panels showed the photographs of several nematodes under UV. Scale bar = 50 μm.
(TIF)

S2 Fig Alignment of amino acid sequences of Dd_02440, Dd_11154 and Dd_13225 in *D*. *destructor*. (TIF)

S3 Fig Detection of the three purified alpha-amylase proteins. (A) The purified proteins were analyzed using SDS-PAGE and stained with Coomassie blue. Lane M: protein marker (26614, Fermentas Thermo); Lane 1: protein of the induced *E*. *coli* cells containing the empty pET-28a vector by 0.1 mM isopropyl-beta-D-thiogalactopyranoside (IPTG); Lane 2: protein of the induced *E*. *coli* cells containing the recombinant pET-Dd_11154 by 0.1 mM IPTG; Lane 3: purified Dd_11154 protein (≈ 160 kDa); Lane 4: protein of the induced *E*. *coli* cells containing the recombinant pET-Dd_02440 by 0.1 mM IPTG; Lane 5: purified Dd_02440 protein (≈ 84 kDa); Lane 6: protein of the induced *E*. *coli* cells containing the recombinant pET-Dd_13225 by 0.1 mM IPTG; Lane 7: purified Dd_13225 protein (≈ 35 kDa). (B) Western blotting analysis of purified alpha-amylase proteins. Lane 1: purified Dd_13225 protein; Lane 2: protein of the non-induced *E*. *coli* cells with pET-Dd_13225; Lane 3: purified Dd_02440 protein; Lane 4: protein of the non-induced *E*. *coli* cells with pET-Dd_02440; Lane 5: purified Dd_11154 protein; Lane 6: protein of the non-induced *E*. *coli* cells with pET-Dd_11154.
(TIF)

S1 Table Primers used in this study. (DOCX)
