# Supplementary figures and images for: Multi-copy alpha-amylase genes are crucial for Ditylenchus destructor to parasitize the plant host
Source: PLoS One. 2020 Oct 26;15(10):e0240805. doi: 10.1371/journal.pone.0240805 (PMC7588122; doi:10.1371/journal.pone.0240805)

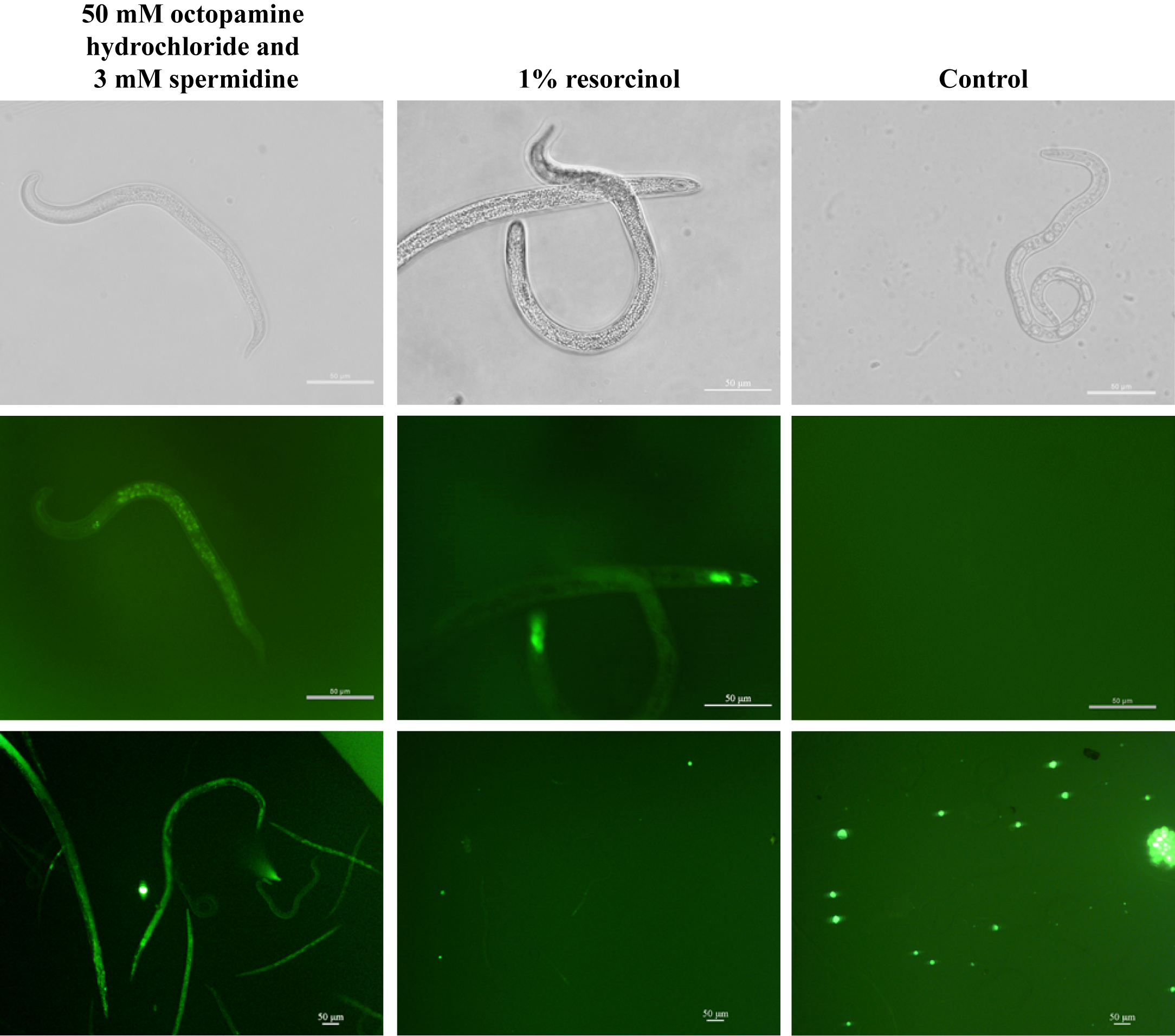

Supplement: S1 Fig — Fluorescence was present in the intestine of nematodes soaked with 50 mM octopamine hydrochloride and 3 mM spermidine. Fluorescence was only present in the stylet of nematodes soaking with 1% resorcinol occasionally, and no fluorescence was observed in nematodes soaked in control solution without any stimulants. The top and middle panels show the photographs of a single nematode on a bright field and under UV, respectively. The bottom panels showed the photographs of several nematodes under UV. Scale bar = 50 μm. (TIF) [file pone.0240805.s001.tif]

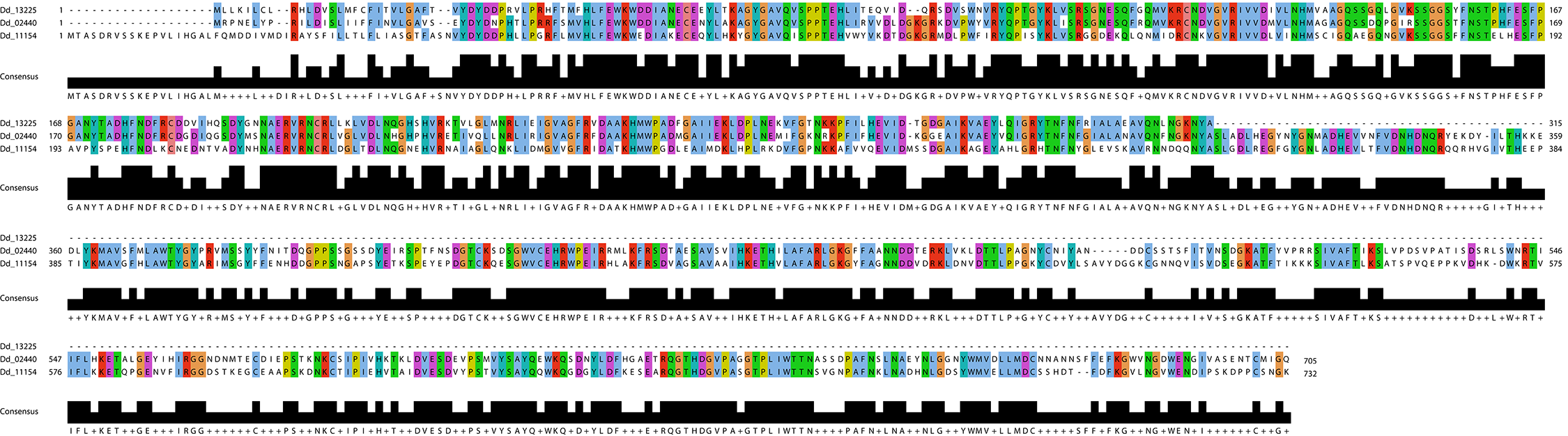

Supplement: S2 Fig — (TIF) [file pone.0240805.s002.tif]

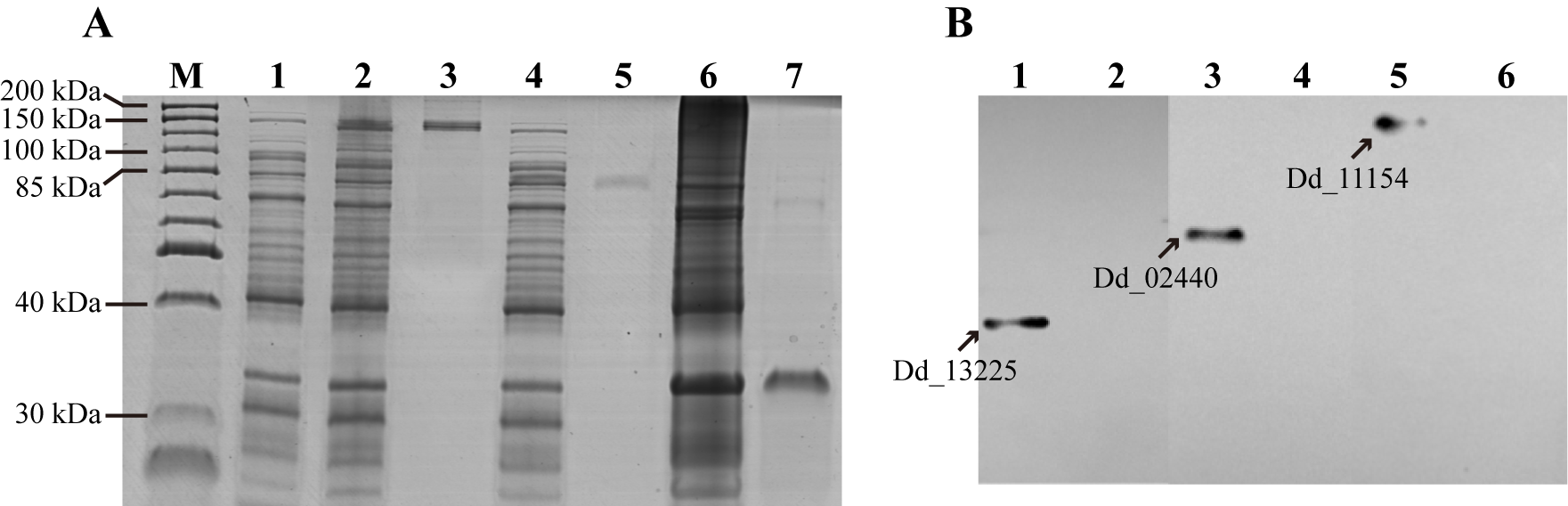

Supplement: S3 Fig — (A) The purified proteins were analyzed using SDS-PAGE and stained with Coomassie blue. Lane M: protein marker (26614, Fermentas Thermo); Lane 1: protein of the induced E. coli cells containing the empty pET-28a vector by 0.1 mM isopropyl-beta-D-thiogalactopyranoside (IPTG); Lane 2: protein of the induced E. coli cells containing the recombinant pET-Dd_11154 by 0.1 mM IPTG; Lane 3: purified Dd_11154 protein (≈ 160 kDa); Lane 4: protein of the induced E. coli cells containing the recombinant pET-Dd_02440 by 0.1 mM IPTG; Lane 5: purified Dd_02440 protein (≈ 84 kDa); Lane 6: protein of the induced E. coli cells containing the recombinant pET-Dd_13225 by 0.1 mM IPTG; Lane 7: purified Dd_13225 protein (≈ 35 kDa). (B) Western blotting analysis of purified alpha-amylase proteins. Lane 1: purified Dd_13225 protein; Lane 2: protein of the non-induced E. coli cells with pET-Dd_13225; Lane 3: purified Dd_02440 protein; Lane 4: protein of the non-induced E. coli cells with pET-Dd_02440; Lane 5: purified Dd_11154 protein; Lane 6: protein of the non-induced E. coli cells with pET-Dd_11154. (TIF) [file pone.0240805.s003.tif]
