# Supplementary material for: Multi-copy alpha-amylase genes are crucial for Ditylenchus destructor to parasitize the plant host
Source: PLoS One. 2020 Oct 26;15(10):e0240805. doi: 10.1371/journal.pone.0240805 (PMC7588122; doi:10.1371/journal.pone.0240805)
Supplement: S1 Table — (DOCX) [file pone.0240805.s004.docx]

**Table S1. Primers used in this study.**

| **Gene** | **Primer** | **Sequence** | **Primer use** |
| --- | --- | --- | --- |
| *Dd_02440* | Dd_02440-qRT-F | AGAATGCGAGCAGTACCTTCACAA | qRT-PCR |
|  | Dd_02440-qRT-R | GCTGATGGGCTGATAACGGATGA |  |
|  | Dd_02440-probe-sense | ACCACGACAATCAACGCTAC | *In situ* hybridization |
|  | Dd_02440-probe-antisense | ATCGGAATTGAAGGTTGGACTT |  |
|  | Dd_02440-cds-F | (CCGGAATTC)^a^ATGCGCCCAAACGAATTA | Clone and expression |
|  | Dd_02440-cds-R | (CCGCTCGAG)TTAATAATAATAGTCATC |  |
|  | Dd_02440-RNAi-F | TAATACGACTCACTATAGGG^b^GCTACGAAAAGGATTACA | dsRNA template |
|  | Dd_02440-RNAi-R | TAATACGACTCACTATAGGGGCGTTTCCTTATGGATAA |  |
| *Dd_11154* | Dd_11154-qRT-F | TTGCTAATGAGTGCGAGAACTACT | qRT-PCR |
|  | Dd_11154-qRT-R | TCCACTTCGGCTAATCAACTTGTA |  |
|  | Dd_11154-probe-sense | TCGTAGGAGTGAATGGAGTGAA | *In situ* hybridization |
|  | Dd_11154-probe-antisense | ATGAGCATCGCCTTGATTGAG |  |
|  | Dd_11154-cds1-F | (CGCGGATCC)ATGACAGCAAGTGATAGA | Clone and expression |
|  | Dd_11154-cds1-R | *TCGTCGTAGTCATAG*^c^ACGTTGTTGAAAATGTTC |  |
|  | Dd_11154-cds2-F | *CATTTTCAACAACGT*CTATGACTACGACGATCC |  |
|  | Dd_11154-cds2-R | (CCGCTCGAG)TCATTTCTTGTCCACAAT |  |
|  | Dd_11154-RNAi-F | TAATACGACTCACTATAGGGGGCACTTTTGCAAGCAAC | dsRNA template |
|  | Dd_11154-RNAi-R | TAATACGACTCACTATAGGGGGCCAATGCAAGACATGT |  |
| *Dd_13225* | Dd_13225-qRT-F | AACCAAGGCAGGCTATGGAG | qRT-PCR |
|  | Dd_13225-qRT-R | CGCTTCGGCTAACCAATTTATACC |  |
|  | Dd_13225-probe-sense | ATTATGCCTCAGACACCTCGAT | *In situ* hybridization |
|  | Dd_13225-probe-antisense | TCCATAGCCTGCCTTGGTTAA |  |

**Table S1. Continued.**

| **Gene** | **Primer** | **Sequence** | **Primer use** |
| --- | --- | --- | --- |
| *Dd_13225* | Dd_13225-cds-F | (CCGGAATTC)ATGTTGCTAAAGATATTATGCCT | Clone and expression |
|  | Dd_13225-cds-R | (CGGCTCGAG)TTACGCATAGTTTTTCCCATTGA |  |
|  | Dd_13225-RNAi-F | TAATACGACTCACTATAGGGGCTAAAGATATTATGCCT | dsRNA template |
|  | Dd_13225-RNAi-R | TAATACGACTCACTATAGGGCCGCTTCGGCTAACCAAT |  |
| *Dd-tba-1* | Dd-tba-1-qRT-F | ACATTCTTCAGTGAGACGCAATC | qRT-PCR internal reference |
|  | Dd-tba-1-qRT-R | ACCTTGGAGACCGTGACATT |  |
| *gfp* | GFPRNAi-F | TAATACGACTCACTATAGGGGAGTGCCATGCCCGAAGG | dsRNA template |
|  | GFPRNAi-R | TAATACGACTCACTATAGGGGGTCTGCTAGTTGAACGC |  |

a: Sequences in brackets are the recognition sequence of restriction enzyme.

b: Underlined sequences are the T7 polymerase promoter.

c: Italicized sequences are sequences for gene splicing by overlap extension (SOE).
